# Occupational exposure to diesel exhausts and pancreatic cancer

**DOI:** 10.1097/JS9.0000000000001498

**Published:** 2024-05-03

**Authors:** Peng Ge, Yalan Luo, Hailong Chen

**Affiliations:** a Department of General Surgery, The First Affiliated Hospital of Dalian Medical University; b Laboratory of Integrative Medicine, The First Affiliated Hospital of Dalian Medical University; c Department of Gastroenterology, The First Affiliated Hospital of Dalian Medical University; d Institute (College) of Integrative Medicine, Dalian Medical University, Dalian, Liaoning, People’s Republic of China

*Dear Editor,*

The cross-sectional study published in the *International Journal of Surgery* by Li *et al*.^1^ piqued our interest because it demonstrated that smoking, high body mass index, and high fasting plasma glucose all increased the risk of early-onset pancreatic cancer (PC). Their results are a foundation for informing public health and prevention strategies about PC. Environmental factors constitute a significant etiology of cancer. For instance, occupational exposure to diesel exhaust (DE) is a risk factor for lung cancer^2^. Nevertheless, the causal relationship between occupational exposure to DE and the risk of PC remains a subject of debate. A previous epidemiological analysis of 26 studies concluded that occupational exposure to DE did not significantly increase PC risk^3^. Sassano *et al*.^4^ discovered, however, in a recent systematic review of 15 studies, that occupational DE exposure marginally increased the risk of PC. The intricate correlation between occupational exposure to DE and PC is examined in this paper.

Although randomized controlled trials (RCTs) are widely regarded as the gold standard for assessing causal effects, their implementation often encounters challenges related to ethics, subject compliance, and study periods. Mendelian randomization (MR) replicates the relationship between exposure and outcome by constructing the ‘instrumental variables (IVs)-outcome’ effect^5^. It employs genetic variation as an IV. Based on the benefits of efficiently eliminating the impact of confounding variables and reverse causal interference, the use of MR in epidemiological research is expanding. This paper investigated the causal relationship between occupational exposure to DE and PC risk using a two-sample MR approach.

The genome-wide association study (GWAS) data of exposure (Workplace had a lot of diesel exhaust: Often) were obtained from the UK Biobank, including 3483 cases and 85 621 controls. From the FinnGen Consortium (R10), GWAS data for outcome (PC), including 1626 cases and 314 193 controls, were obtained. Genetic variation associated with exposure was filtered in light of three fundamental hypotheses. To ensure the inclusion of a maximum number of single nucleotide polymorphisms (SNPs), we lowered the significance threshold to an acceptable level (*P*-value below 5E−06). The parameter for linkage imbalance is established with *r* ^2^ below 0.0001, and the genetic distance is set to 10 000 kb. Furthermore, SNPs with effect allele frequencies exceeding 0.42 and *F* statistics below 10 are eliminated, as detailed in Supplementary Table S1 (Supplemental Digital Content 1, http://links.lww.com/JS9/C457). The primary approach for MR analysis is inverse variance weighting (IVW), further supported by the weighted median, simple mode, MR-Egger regression, and weighted model methods. *P* values were computed utilizing IVW (Cochran’s *Q* test) to evaluate heterogeneity. Egger intercept was implemented to examine the pleiotropy. MR-pleiotropy residual sum and outlier were used to identify the presence of outliers in the selected SNPs. The statistical analysis was conducted using the R 4.3.0 software and the MR-PRESSO and TwoSampleMR packages.

An IVW analysis indicated that occupational exposure to DE lacks a causal association with the risk of PC (odds ratio=0.166, 95% confidence interval=0.003–7.923, *P*=0.362). The extrapolation of the IVW indicated that the effect magnitude and orientations of the four complementary approaches were consistent (Supplementary Figure S1, Supplemental Digital Content 2, http://links.lww.com/JS9/C458). No heterogeneity was detected by Cochran’s *Q* test (*I* ^2^=1.529%, *P*=0.432). No pleiotropy was identified by MR-Egger regression (egger intercept=0.012, *P*=0.715). A global MR-PRESSO test yielded no outliers (*P*=0.344). The inference of this MR analysis can be characterized as dependable.

Referred to as the ‘natural RCT,’ the MR method aims to minimize the influence of confounding factors and reverse causation. This is an advantageous aspect of this analysis. However, the proportion of exposure variation that IVs can account for is a limitation. A single SNP plays a negligible role in elucidating variation in exposure and should not be equated with exposure. Despite lowering the significance threshold to incorporate a more significant number of SNPs, the accuracy of estimation and statistical power may still need to be improved. As a result, additional GWAS data is required to clarify the molecular mechanisms underlying these connections.

This study concludes that occupational exposure to DE does not provide sufficient evidence to establish a causal relationship with PC.

## Ethical approval

Not applicable. The GWAS concerning exposure and outcomes were derived from publicly accessible databases previously sanctioned by ethical approval, thereby preventing the necessity for additional ethical clearances in the context of this study.

## Consent

Not applicable.

## Sources of funding

This study was supported by the National Natural Science Foundation of China (Nos. 82274311 and 82074158) and the National Key R&D Program of China (No. 2019YFE0119300).

## Author contribution

P.G. and Y.L.: contributed equally to this work; H.C.: conceptualization and funding acquisition; P.G.: methodology and writing – original draft; Y.L.: writing – review and editing.

## Conflicts of interest disclosure

All authors disclose no conflicts of interest in this study.

## Research registration unique identifying number (UIN)

Not applicable.

## Guarantor

Hailong Chen.

## Data availability statement

The genome-wide association study (GWAS) summary data, which provide evidence for the conclusions drawn in this research, may be accessed publically via the UK Biobank study and the FinnGen database.

## Provenance and peer review

Our paper was not invited.

## Supplementary Material
