# Supplementary figures and images for: Occupational exposure to diesel exhausts and pancreatic cancer
Source: Int J Surg. 2024 May 3;110(8):5226–7. doi: 10.1097/JS9.0000000000001498 (PMC11325946; doi:10.1097/JS9.0000000000001498)

# MR Test

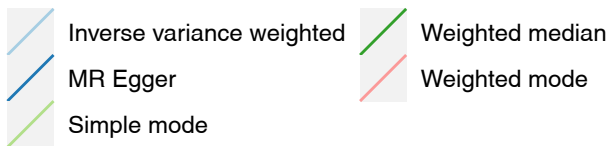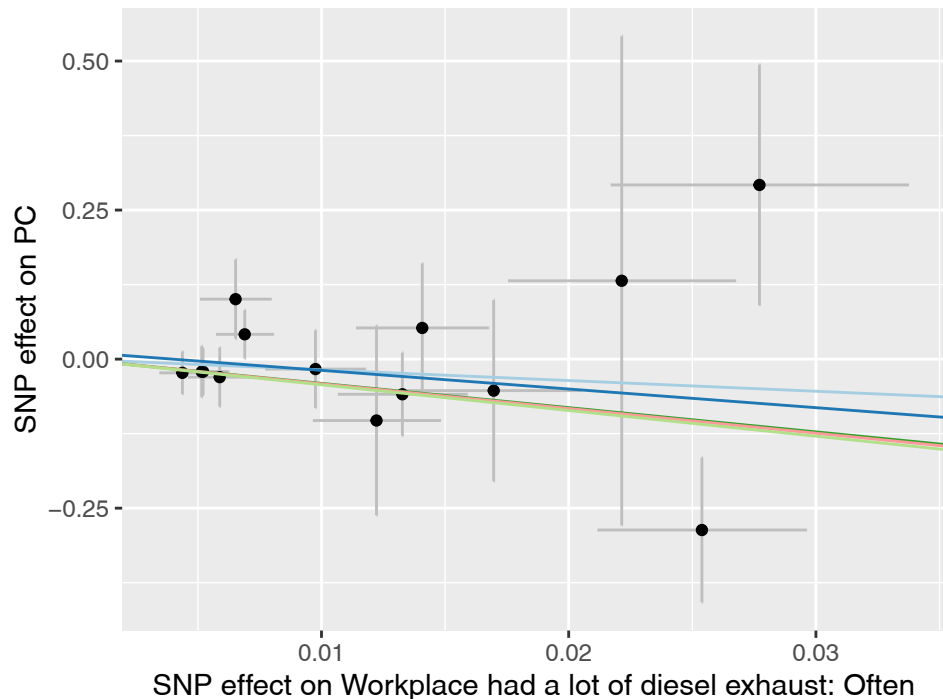

Figure S1. Scatter plot of MR analysis.

Supplement: Supplementary file 2 [file js9-110-5226-s002.pdf]
